# The Roles of Siglec7 and Siglec9 on Natural Killer Cells in Virus Infection and Tumour Progression

**DOI:** 10.1155/2020/6243819

**Published:** 2020-04-06

**Authors:** Yayun Zheng, Xue Ma, Dongmei Su, Yue Zhang, Lin Yu, Fangfei Jiang, Xue Zhou, Ying Feng, Fang Ma

**Affiliations:** ^1^Center for Translational Medicine, Key Laboratory of Birth Defects and Related Diseases of Women and Children (Sichuan University), Ministry of Education, West China Second University Hospital, Sichuan University, Chengdu, Sichuan 610041, China; ^2^Department of Obstetrics and Gynecology, West China Second University Hospital, Sichuan University, Chengdu, Sichuan 610041, China; ^3^Department of Histology, Embryology and Neurobiology, West China School of Basic Medical Sciences & Forensic Medicine, Sichuan University, Chengdu, Sichuan 610041, China; ^4^Department of Pediatric Urology, West China University Hospital, Sichuan University, Chengdu, Sichuan 610041, China

## Abstract

The function of natural killer (NK) cells, defending against virus infection and tumour progression, is regulated by multiple activating and inhibiting receptors expressed on NK cells, among which sialic acid-bind immunoglobulin-like lectins (Siglecs) act as a vital inhibitory group. Previous studies have shown that Siglec7 and Siglec9 are expressed on NK cells, which negatively regulate the function of NK cells and modulate the immune response through the interaction of sialic acid-containing ligands. Siglec7 and Siglec9 are very similar in distribution, gene encoding, protein sequences, ligand affinity, and functions in regulating the immune system against virus and cancers, but differences still exist between them. In this review, we aim to discuss the similarities and differences between Siglec7 and Siglec9 and analyze their functions in virus infection and tumour progression in order to develop better anti-viral and anti-tumor immunotherapy in the future.

## 1. Introduction

Natural killer (NK) cells are essential innate immune cells which are able to directly kill unhealthy host cells, including virus-infected cells [1] and tumour cells [2]. Due to the downregulation of MHC I of virus-infected cells and tumour cells, the target cells can escape from the specific recognition of T cells [3]. However, NK cell-mediated cytotoxicity does not require antigenic stimulation and is not restricted by MHC I molecules. Various activating receptors, such as NKp30, NKp44, NKp46, CD16, NKG2C, and NKG2E, are involved in the NK-mediated cytotoxicity. Not only activating receptor interaction with ligands can induce NK cells to kill target cells but also inhibitory receptors take part in regulating the cellular cytotoxicity exerted by NK cells.

Siglecs are important fragments of receptors, which regulate inhibitory signal conduction on NK cells [4]. They are characterized subset of immunoglobulin superfamily and one family of type I transmembrane proteins of I-lectin. Sialic acid (Sia), on the one hand, is a group of 9-carbon-backnone monosaccharide and is necessary for immunomodulation; on the other hand, it is hydrophilic and negatively charged and widely expressed at the terminal of the protein or lipid on the cell surface and secretory proteins [5, 6]. Siglecs are critical receptors of Sia. Siglecs promote cell-cell recognition and modulate the cytotoxicity of NK cell towards virus and tumour by binding to the Sia residue of the glycoconjugates on target cells. According to the evolution, Siglecs can be divided into two groups. The conserved Siglecs include sialoadhesin (Siglec1), CD22 (Siglec2), MAG (Siglec4), and Siglec15. Another group involves a group of rapidly evolving Siglecs, named CD33-related Siglecs. In human, CD33-related Siglecs consist of CD33 (Siglec3), Siglec5, Siglec6, Siglec7, Siglec8, Siglec9, Siglec10, Siglec11, Siglec14, and Siglec16. However, due to no homology between human and mouse, CD33-related Siglecs in mouse comprise CD33 (Siglec3), Siglec-E, Siglec-F, Siglec-G, and Siglec-H.

Siglecs play important roles in mediating immune response. For example, CD22 (Siglec-2), specifically expressed on B cells, interacts with its ligand, *α* 2,6-linked Sia on adjacent cells, inhibiting cell activating signal transduction, calcium mobilization and B cell receptor activation [7]. Besides, antibody blocking of Siglec8 can induce caspase-3-like apoptosis and inhibit eosinophil viability [8]. Moreover, the interaction between Siglec7 and Sia,or between Siglec9 and Sia leads to the phosphorylation of immunoreceptor tyrosine-based inhibition motif (ITIM), which can suppress the cytotoxicity of NK cells. Whereas the most prominent Siglecs of immune regulation on NK cells are Siglec7 and Siglec9 [9], this review will focus on the current research progress on the similarities and differences between Siglec7 and Siglec9 and their functions in tumour and virus infection progression.

## 2. The Distribution of Siglec7 and Siglec9

Siglec7 and Siglec9 are mainly expressed in immune cells. Siglec7 is primitively reported to be expressed on NK cells, especially in CD56^bright^ cells. In addition, a minor subset of CD8^+^ T cells, representing cytotoxic T cells, was also confirmed to express Siglec7. The expression level in granulocyte was reported to be lower than monocyte, which was lower than the lymphocyte's [10]. In the past years, it was also proved that Siglec7 was expressed on macrophages [11], dendritic cells [12], mast cells, and basophils [13] (Table 1). Siglec9 is expressed quite broadly among human blood leukocytes, including monocytes, neutrophils, B cells, NK cells, and minor subsets of T cells [14]. In peripheral blood, the expression of Siglec9 is predominantly on neutrophils [15–17] and followed by NK cells, B cells, and monocytes [11]. Siglec9 was also detected on tissue resident macrophages at low level [11, 18, 19] (Table 1). And it was reported that the Siglec9 level on neutrophil rose higher than adults during the neonatal period, but the high level withdrew in one month after birth. This may be caused by the infection of the bacterium, Group B *Streptococcus* (GBS), presenting mostly in neonatal period [20]. Although the cDNA of Siglec9 was cloned from promyeloblast, HL60, little evidence showed that HL60 cells express the protein of Siglec9. It indicates that Siglec9 is possibly expressed in advanced differentiating immune cells [18].

Apart from the immune system, Siglec7 is also found in other cells, while the expression of Siglec9 in other cells has not been reported. Nguyen et al. illustrated that Siglec7 was detected on platelets of healthy donators, and the cross-linking Siglec7 with its ligand, ganglioside, resulted in platelet apoptosis [21]. Therefore, Siglec7 may be a potential therapy target of platelet diseases. Moreover, Dharmadhikari et al. revealed that Siglec7 detected in *β*-cells of human pancreatic islets may participate in glycan metabolism by inhibiting *β*-cell apoptosis [22].

## 3. The Gene and Protein Sequence Comparison of Siglec7 and Siglec9

**Figure 1:**
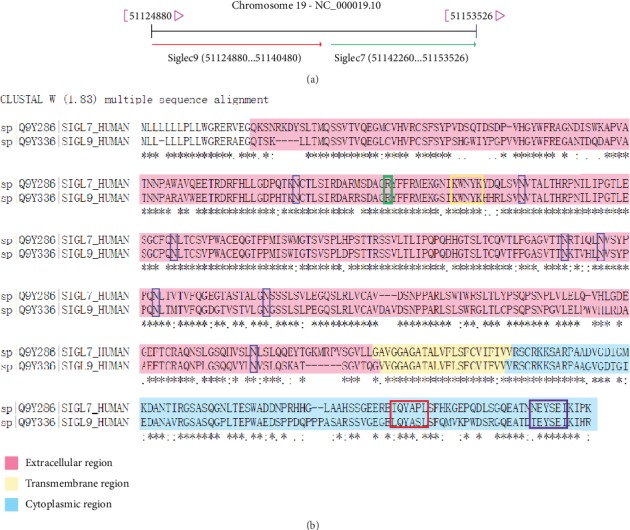
(a) The gene location of Siglec7 and Siglec9. They are both mapped in the chromosome 19q13.3-13.4. (b) The sequence alignment of protein Siglec7 and Siglec9. The protein sequences are from uniport (The UniProtKB of Siglec7 Human: Q9Y286; Siglec9 Human: Q9Y336). The topological domains are colored in pink, light yellow, and blue, representing the extracellular region, transmembrane region, and cytoplasmic region, respectively. Asterisk: indicating a conserved amino acid in all sequences; colon: indicating the position of a sequence alignment composed of residues having similar physicochemical properties; point: indicating the column of the multiple sequence alignment in which the semiconservative substitution was observed. The dark blue frames indicate the eight glycosylation sites. The green frame represents the Sia binding site, and the yellow frame indicates the Sia binding region. The red frame denotes the ITIM motif, and the purple frame signifies the ITIM-like motif.

With around 84% identity in coding sequence, Siglec7 (p75/AIRM1 or CD328) and Siglec9 encoding genes are both located in chromosome 19q13.3-13.4 (Figure 1(a)). Full-length cDNA sequence of Siglec7 was firstly cloned from a human primary dendritic cell cDNA library by Nicoll and his colleagues in 1999, and it is 1748 base pair long encoding a 467-amino acid protein [10]. While Siglec9's full-length cDNA sequence was found in the cDNA library of human acute promyelocytic leukemia cells one year after Siglec7, which contain a 1392-nucleotide open reading frame and produces a 463-amino-acid protein [23].

The proteins of Siglec7 and Siglec9 are both type I membrane proteins of the Ig superfamily. Siglec7 is a 75 kDa protein (the monomer of Siglec7 is reported to be about 65 kDa) [10], while the protein of Siglec9 is 50.1 kDa [23]. Although there is a difference of molecular weight between Siglec7 and Siglec9, the protein structure of Siglec7 is very similar to that of Siglec9 (Figure 1(b), Table 2). They both consist of an extracellular region, a transmembrane region, and a cytoplasmic tail. The extracellular region of both Siglec7 and Siglec9 contains a hydrophobic signal peptide and three Ig-like domains including one N-terminal V-set domain and two C2-set domains. The hydrophobic signal peptide in the extracellular region has 18 amino acids in Siglec7 and 17 amino acids in Siglec9, respectively. In the three Ig-like domains of both Siglec7 and Siglec9, there are eight potential N-linked glycosylation sites [10, 14]. Whereas when Siglec7 was treated with *N*-glycosidase, it showed a protein backbone of 48 kD [24].Except for the N-linked glycosylation sites, the Sia binding sites exist in both of the proteins. The binding of Siglecs with Sia requires multiple sites to function simultaneously (please see Section 4 below for details).

Although Siglec7 and Siglec9 are reported as type I transmembrane proteins, Varchetta et al.'s group discovered a soluble Siglec7 (sSiglec7) without a transmembrane region in circular peripheral blood [25]. Zeng et al.'s group also unveiled a soluble Siglec9 (sSiglec9) in the plasma, which can induce the oxidative stress, and the expression can be increased by TNF-*α*, IL-6, and IL-8 [26]. Matsumoto et al.'s group also detected the injected-sSiglec9 in the inflamed tissue of the mouse paws and all digits after intravenously administering recombinant human Siglec9 into arthritis mice. They also proved that sSiglec9 can suppress arthritis in the mouse model [27]. Siglec-E is the mouse ortholog of human Siglec-9. However, whether Siglec-E participated in suppressing arthritis was not mentioned in the study. While human sSiglec9 transgenic mice presented resistance against GBS infection [28], as well as anti-tumour benefit against mammary tumour cells [29], the structure of sSiglec9 has yet not been well characterized.Hence, further study is required.

The cytoplasmic regions of Siglec7 and Siglec9 both have two important functional domains: a membrane proximal ITIM domain and a membrane-distal motif. Pro^439^ in the proximal motif and Asn^458^ in the distal motif of Siglec7 both contribute to recruiting phosphatases [30]. The ITIM motif in Siglec9 is very similar to Siglec7, Ile^435^-Gln-Tyr-Ala-Pro-Leu^440^ in Siglec7 and Leu^431^-Gln-Tyr-Ala-Ser-Leu^436^ in Siglec9. And the membrane-distal motif of Siglec9 Thr^454^-Glu-Tyr-Ser-Glu-Ile^459^ is an ITIM-like motif, which is Asn^458^-Glu-Tyr-Ser-Glu-Ile^463^ in Siglec7 [10, 18].

## 4. The Ligand Affinity of Siglec7 and Siglec9

Although comparison of the amino acid sequences between Siglec7 and Siglec9 results in a similarity up to 98% (comparison tool: T-coffee http://tcoffee.vital-it.ch/), ligand affinity differences still exist between Siglec7 and Siglec9. It was reported that Siglec7 binds to *α*2,8-sialyl residue with the highest affinity, while binds to *α*2,6-sialyl residue with lower affinity and *α*2,3-sialyl residue with the lowest affinity [31, 32]. Meanwhile, Siglec9 prefers to bind to *α*2,6-sialyl and *α*2,3-sialyl residues [14, 31].

Siglec7 and Siglec9 can also bind to sulfated sialyl Lewis^x^, which was proved on normal colonic epithelial cells. And the expression of sulfated sialyl Lewis^x^ decreased when these cells become colonic cancer cells. The ligand affinity was confirmed by a consortium of functional glycomics using ELISA on molecular level (http://www.functionalglycomics.org) and flow cytometric analyses on cell level in Miyazaki et al.'s group [11]. The ELISA results showed that Siglec-7 was the seventh reactive to sialyl 6-sulfo Lewis^x^, while Siglec9 was the most reactive. However, cell level results showed that sialyl 6-sulfo Lewis^x^ was detected strongly binding to Siglec7 and hardly binding to Siglec9. The contradictory findings may be due to that ELISA results on a molecular level may not always be consistent with the cell experiments, and this requires further study.

The Sia binding characteristics of Siglec7 and Siglec9 are determined by specific amino acids. Yamaji et al.'s group exchanged the V-set domain of Asn (70)-Lys (75) between Siglec7 and Siglec9, resulting in loss of the original binding specificities and gaining of each other's binding properties. Proved by aforementioned evidence, they further highlighted that the affinity specificity of Siglec7 and Siglec9 was determined by the C-C loop of the glycan binding domain [33], whereas the Arg124 in Siglec7 and Arg120 in Siglec9 was the definite amino acid for binding to Sia. It was reported that Siglec-9 transiently transfecting to COS cells was able to recognize red blood cells, but COS cells expressing Arg120-mutant Siglec9 could not bind to red blood cells. This result indicated that the stable salt bridge connecting Arg and Sia is necessary for binding [18, 31].

Moreover, Malaker et al. proved that cells treated with secreted protease of C1 esterase inhibitor (StcE) had a decreased affinity with Siglec7, but the binding to Siglec9 was not affected. StcE is a bacterial protease from *E. coli* and selective for mucin-domain cleavage by identifying discrete peptide- and glycan-based motifs. The main feature of the mucin domain is the high frequency of Ser and Thr residues that are *O*-glycosylated by *α*-*N*-acetylgalactosamine (*α*-GalNAc) [34]. This phenomime indicates that the binding between Siglec7 and cells depends on the mucin domain, and the binding between Siglec9 and cells may rely on some other motifs rather than mucin domain. Besides, StcE was reported to mainly cut densely *O*-glycosylated proteins and has no reactivity with *N*-glycosylated proteins and sparsely *O*-glycosylated [35]. The difference in ligand affinity between Siglec7 and Siglec9 may not only be due to the differences in sialylation site but also be due to the differences in *N*-glycosylation or *O*-glycosylation, even the density of glycosylation. Taken together, accumulating evidences indicate Siglec7 and Siglec9 have diverse ligand affinity which, to some extent, explains the different function of the two lectins in immune cells.

## 5. The Function of Siglec7 and Siglec9 in NK Cells

Siglec7, Siglec9, and Siglec-17 are expressed in NK cells and play important roles in inhibitory signal transducing through Sia-dependent binding. Siglec7, first detected in NK cells in 1999, serves as an inhibitory receptor mediating Sia-dependent ligand recognition [10, 24]. Shao et al. proved that the Siglec7^+^ subgroup of NK cells produces more CD107a degranulation and secretes more cytokines, such as IFN-*γ* and TNF-*α*, and they considered Siglec7 as a marker of a higher function group of NK cells [36]. Siglec9, which is highly related to Siglec7, was first found in NK cells in 2000 [14, 18]. It has been proved that the expression of Siglec9 happened in the early stage of NK cell differentiation from CD56^bright^ to CD56^dim^. Jandus et al. indicated that Siglec9 might be a maker for an early maturity group of NK cells, which are less cytotoxic but more chemotactic [9]. It was previously reported that Siglec7 was expressed on all NK cells [10, 24]. However, more and more evidences show that there are Siglec7^−^ NK cell subsets [10, 36, 37]. Shao et al. reported that Siglec7 was preferentially expressed on mature NK cells, and Siglec7^+^ NK cells express more activating receptors and less inhibitory receptors. They believed that Siglec7 may be defined a more cytotoxic group of NK cells [36]. And Siglec9 is expressed on 30-40% of CD56^dim^ NK cells [19] and shows very weakly positive on CD56^bright^ NK cells [18]. Except for immune function, it is showed that the expression of Siglec7 decreased on NK cells among the obese population (BMI > 30 kg/m^2^), which was not applicable to Siglec9 [38]. However, whether Siglec7 participates in lipid metabolism remains unclear.

**Figure 2:**
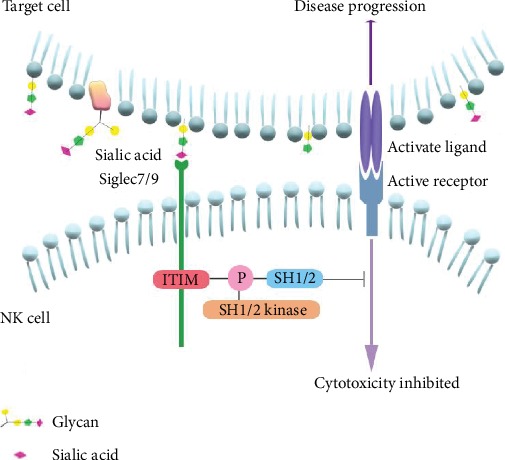
Siglec-7/9, cross-talking with sialic acids (Sia) on the surface of target cells, can inhibit the cytotoxicity of NK cells. The interaction between siglec7/9 and Sia can lead to the phosphorylation of ITIM and recruit SHP-1 and SHP-2, which will suppress the NK cell activation, and resulting in disease progression.

Abundant evidences have proved that Siglec7 and Siglec9 exhibit inhibitory roles in regulating the immune balance [39, 40]. The important functional domain of both Siglecs7 and Siglec9 is the membrane-proximal ITIM, mediating the inhibitory signaling. Siglecs crosstalk with Sia of the glycoconjugates through its N-terminal Sia-binding domain and mediate cell-cell recognition, which induce tyrosine phosphorylation. ITIMs are able to provide docking sites for Src homology 1/2domain containing cytoplasmic phosphatases(SHP-1/2) when tyrosine phosphorylated, which is critical for signal delivery [41, 42]. Through the interaction with Sia, Siglec7 can reduce the cytotoxicity of NK cells towards the target cells through the ITIM recruiting SHP-1/2, blocking signal conduction pathway, and playing negative regulation (Figure 2) [43]. Here, we aim to discuss the functions of Siglec7 and Siglec9 in virus infection and tumour progression.

### 5.1. Siglec7 Involves in Human Immunodeficiency Virus-1 (HIV-1), Hepatitis B Virus (HBV), and Hepatitis C Virus (HCV) Infection, While Siglec9 Participates in the HBV Replication

Siglec7 is involved in HIV-1 infection, which usually accompanies the changes of Siglec7 expressions on NK cells while maintaining the total number of NK cells in the peripheral blood [44, 45]. Brunetta et al.'s group indicated that Siglec7 may be a biomarker of disorder functional subsets of NK cells and HIV-1 infection, because they found that reduced number of Siglec7^+^ NK cell subgroup was related to the high level of HIV-1 replication [44]. They also believed that the downregulation of Siglec7 on NK cells was due to the decreased size of the Siglec7^+^ NK cell subgroup and increased amount of the Siglec7^−^ NK cells, while the total number of NK cells in the peripheral blood was not reduced. Moreover, based on the study of Siglecs endocytic function [46], Brunetta et al. provided another hypothesis that Siglec7 may bind to HIV-1 envelope (Env) glycoprotein 120 (gp120), triggering the endocytic process of siglec7 [45]. Zulu et al.'s group discovered a decrease of Siglec7 on NK cells in most of HIV-1 samples from the peripheral blood collected from healthy donors, chronic viremic, long-term nonprogressor (LTNP), and early viremic HIV-1-infected patients. They also found out that Siglec7 in CD56^dim^ NK cells in viremic patients decreased with comparison to healthy donors [47], whereas Brunetta et al.'s group proved that antiretroviral therapy can rescue the expression of Siglec7 [44]. And *in vitro* studies showed that recombinant human Siglec7 bound to sialyl residues of HIV-1 envelope (Env) glycoprotein 120 (gp120), which mediated virus entrying into target cells [25, 48]. It revealed that Sia residues on gp120 can protect the virus through Siglec7 from the human immune system attacking and promote virus replication and disease progression. However, according to Varchetta et al.'s group, an increase level of serum sSiglec7 was detected in AIDS patients compared to healthy donors [25]. Siglec7 on the NK cell surface was suggested to be masked by virus, and the serum sSiglec7 may be from the apoptosis NK cells [25, 44, 49].

Besides, Siglec7 may contribute to HCV and HBV infection. Similar to the expression profile in HIV-1 patients, the expression of Siglec7 on NK cells declined in HCV-infected and HBV-infected patients, whereas the serum sSiglec7 level increased [37, 50]. The serum sSiglec7 level was associated with the HBV/HCV level and negatively correlated with Siglec7^+^ NK cells.

Siglec9 participates in the hepatitis B virus (HBV) replication and is involved in the dysfunction of NK cells. Zhao et al.'s group pointed out that the level of Siglec9 on NK cells in HBV-infected patients decreased. And the expression level of Siglec9 was negatively correlated with virus replication. Moreover, the Siglec9^+^ NK cells of HBV-infected patients show a higher level of several activating receptors than the Siglec9^−^ NK cells do. However, blocking Siglec9 on NK cells of HBV-infected patients can increase IFN-*γ*, TNF-*α* secretion, and CD107a degranulation [51]. And the expression profile of Siglec9 has not been reported in the infection process in HIV and HCV so far. It would be interesting to analyze the glycol-structure differences between these viruses to reveal the role of Siglec7 and Siglec9 in the above viral infection.

### 5.2. Siglec7 and Siglec9 Binding to Sia Helps Cancer Cells Escape from Immune Surveillance

One of the hotspots in Siglec7/9 researches is their functions in the interaction of cancer cells and NK cells, due to its anti-tumour effects. NK cells participate in killing tumour cells through the specific lysis of target cells and the secretion of cytokine, such as IFN-*γ* and TNF-*α*. Siglec7 and Siglec9 expressed on NK cells and their ligands play important roles in promoting the process of proliferation and migration of tumour cells. The ligands recognized by recognition Siglec7 and Siglec9, Sia, is the glycan chain ending residue of glycolipids and glycoproteins. We introduce the specific roles of Siglecs binding with Sia from the following three aspects: the total Sia: the sialy-glycolipids, ganglioside, and mucins (MUC), a family of heavily sialylated glycoproteins.

Firstly, Sia, the ligand of Siglec7 and Siglec9 expressed on the surfaces of tumour cells, is correlating with the immune evasion in cancer [52]. Jandus et al.'s group reported that Sia was widely expressed on a great number of different organizational tumour cells, which can protect tumour cells from NK cell-mediated cytotoxicity. As Hudak et al.'s group claimed, Sia-equipped glycan can protect tumour cells from the immune system attacking through Sia-Siglec7 interaction [40]. It is worth mentioning that the hypersialylation tumour cells are capable of binding to the Siglec9 on NK cells and modulating immunosurveillance. Jandus et al.'s group also revealed that Siglec9^+^ NK cells expressed more inhibitory receptors (KIR and ILT2) and exhibited a less cytotoxicity towards tumour cells. Treating tumour cells with neuraminidase enhanced the NK cell lysis through the degranulation and the secretion of cytokines [9]. Pearce and Laubli have researched one of the main forms of Sia expressed in mammals: the *N*-glycolylneuraminic acid (Neu5Gc) on the surfaces of tumour cells. Neu5Gc cannot be synthesized by humans, because the human body is lacking of CMP-N-acetylneuraminic acid hydroxylase (the enzyme used to transfer CMP-Neu5Gc from CMP-N-acetylneuraminic (CMP-Neu5Ac) acid [53]). But Neu5Gc can be absorbed from the food and becomes the materials of glycan for the human [54]. Moreover, Neu5Gc can enhance the inflammatory response induced by anti-Neu5Gc [55–57].

Secondly, Siglec7 can interact with ganglioside which is composed of glycosphingolipid with a group of Sia. Kawasaki et al.'s group reported that Siglec7 on NK cells bound to one of the major gangliosides expressed on the surface of renal cell carcinoma (RCC) cells: the disialosyl globopentaosylceramide (DSGb5), which lead to a reduction of NK cell cytotoxicity towards RCC cells. DSGb5 then further promoted RCC metastasis and migration potential [58]. In addition to DSGb5, Siglec7 also interacts with GD3, a ganglioside expressed on tumour cells. Siglec7-GD3 interaction suppresses the NK cell killing activity. Thus, with its highly expressed GD3 ganglioside, melanoma is deemed to escape from NK cell cytotoxicity. And through the transfection of GD3 synthase to the cell lines without the expression of recombinant Siglec7-Fc protein ligands, the cytotoxicity of NK cells to mastocytoma, P815, and colorectal adenocarcinoma cell, DLD-1, was inhibited [43, 59].

Thirdly, Siglec9 can interact with transmembrane epithelial MUC, the heavily glycosylated proteins mainly produced by epithelial tissues, such as MUC1 and MUC16, which leads to immune evasion modulation. MUC1 is overexpressed on adenocarcinomas and hematological cancers [60–62]. MUC1 can induce the growth of tumour cells through recruiting *β*-catenin binding to its C-terminal domain [63]. MUC1-sialylated O-linked glycans on tumour cells binding to Siglec9 did not recruit SHP-1 or SHP-2 but induced calcium flux that lead to the activation of MEK-ERK kinases [64]. Similar to MUC1, Siglec9 can interact with MUC16 expressed on epithelial ovarian cancer cells, protecting tumour cells from immune attacking. Siglec9 promoted tumour cell adhesion process through the recognition of MUC16 glycans which contain *α*2,3-linked Sia, the ligand of Siglec9 [19].

The fact that tumour cells escape from NK cell attack through the interaction of cell surface sialyl-decoration with Siglec7 and Siglec9 on NK cells makes the two lectins promising target in antitumor drug development. Recently, high-affinity low molecular weight Siglec7 ligands are artificially designed and synthesized. The synthetic ligands can weaken the interactions between Siglec7 and its tumour ligands and, hence, inhibit cancer immune evasion [40, 65].

## 6. Conclusion and Outlook

Although Siglec7 and Siglec9 show high similarities in the distribution, gene encoding, protein sequences, ligand affinity, and functions, differences still exist between them. The study of Siglec7 expressed on NK cells is clearer, but the study of Siglec9 on NK cells is lacking. Although Siglec7 and Siglec9 are usually believed to play negative roles in mediating lysis and cytokine secretion in NK cells through Siglecs-ligand recognition, an active role of Siglec7 has been reported as the cross-linking of Siglec7 by a specific antibody that can upregulate the inflammatory cytokines and chemokines in monocytes [66], whereas little research has indicated the active function of Siglec9. With this regard, whether Siglec7 and Siglec9 possess a positive property in immune response and how they play active roles in regulating immune system require further confirmation.

Siglec7 was proved to be related to HIV, HBV, and HCV infection, but only the function of Siglec9 in HBV was reported. The evidence that Siglec9 expressed on NK cells is involved in the immune response to HIV and HCV infection needs further study. There may be structural differences of glycan ligands among HIV, HBV, and HCV, resulting in insufficient recognition of HIV and HCV by Siglec9. Although there are abundant evidences that show Siglec7 and Siglec9 expressed on NK cells are related to the HIV, HBV, and HCV infection, the exact mechanism of Siglec7 and Siglec9 involve in these viral infections requires further investigation.

Siglec7 and Siglec9 binding to the ligands helps the target cells to inhibit the NK cells and also evade immune surveillance. In antivirus and antitumour immunotherapy, the inhibitory effect of Siglec7 and Siglec9 can be blocked by either adding antibodies of the two proteins or the elimination of cell surface ligands of the two. In addition, the regulatory molecules of Siglecs and sialylation can be considered. A glycocalyx engineering approach is created to help tumour cells suppress NK cell lysis. However, whether it contributes to defecting pathogen-infected or tumour deterioration *in vivo* demands further studies.

## Figures and Tables

**Table 1:** The expression and ligand affinity of Siglec7 and Siglec9 in human immune cells.

	Expression									Ligand affinity
	Neutrophil	NK cells	T cells	B cells	Monocyte	Macrophage	Eosinophil	Basophils	Dendritic cells	
Siglec7	+	+	+	-	+	+	+	+	+	*α*2,8>*α*2,6>*α*2,3
Siglec9	+	+	+	+	+	+	-	-	+	*α*2,6 *α*2,3 sulfated residues

**Table 2:** Amino acid sequence analysis of Siglec7 and Siglec9 in human immune cells.

	Signal peptide	Extracellular region			Transmembrane region	Cytoplasmic region	
		Ig-like V-type	Ig-like C2-type 1	Ig-like C2-type 2		ITIM motif	ITIM-like motif
Siglec7	1–18	39–122	150–233	240–336	354–376	435–440	458-463
Siglec9	1-17	20–140	146–229	236–336	349–369	431–436	454-459
